# One Health approach in the prevention and control of mycobacterial infections in Tanzania: lessons learnt and future perspectives

**DOI:** 10.1186/s42522-019-0002-1

**Published:** 2019-11-27

**Authors:** Bugwesa Z. Katale, Erasto V. Mbugi, Julius D. Keyyu, Robert D. Fyumagwa, Mark M. Rweyemamu, Paul D. van Helden, Hazel M. Dockrell, Mecky I. Matee

**Affiliations:** 1 Department of Microbiology and Immunology, School of Medicine, Muhimbili University of Health and Allied Sciences (MUHAS), Dar es Salaam, Tanzania; 2 Tanzania Wildlife Research Institute (TAWIRI), Arusha, Tanzania; 3 Southern African Centre for Infectious Diseases Surveillance (SACIDS), Sokoine University of Agriculture (SUA), Chuo Kikuu, Morogoro, Tanzania; 4 Department of Biochemistry, School of Medicine, Muhimbili University of Health and Allied Sciences (MUHAS), Dar es Salaam, Tanzania; 5 DST/NRF Centre of Excellence for Biomedical Tuberculosis Research/ South African Medical Research Council (MRC) Centre for Tuberculosis Research, Division of Molecular Biology and Human Genetics, Faculty of Health Sciences, Stellenbosch University, Tygerberg, Cape Town, South Africa; 6 Department of Immunology and Infection, London School of Hygiene and Tropical Medicine (LSHTM), London, UK

**Keywords:** One health, Mycobacterial infection, Human-animal-environment, Tanzania

## Abstract

**Background:**

One Health (OH) is an integrated approach, formed inclusive of using multiple disciplines to attain optimal health for humans, animals, and the environment. The increasing proximity between humans, livestock, and wildlife, and its role in the transmission dynamics of mycobacterial infections, necessitates an OH approach in the surveillance of zoonotic diseases. The challenge remains as humans, livestock, and wildlife share resources and interact at various interfaces. Therefore, this review explores the potential of the OH approach to understand the impact of mycobacterial infections in Tanzania in terms of lessons learnt and future perspectives.

**Materials and methods:**

Available literature on OH and mycobacterial infections in Tanzania was searched in PubMed, Google Scholar, and Web of Science. Articles on mycobacterial infections in Tanzania, published between 1997 to 2017, were retrieved to explore the information on OH and mycobacterial infections.

**Main body:**

The studies conducted in Tanzania had have reported a wide diversity of mycobacterial species in humans and animals, which necessitates an OH approach in surveillance of diseases for better control of infectious agents and to safeguard the health of humans and animals. The close proximity between humans and animals increases the chances of inter-specific transmission of infectious pathogens, including drug-resistant mycobacteria. In an era where HIV co-infection is also the case, opportunistic infection by environmental non-tuberculous mycobacteria (NTM), commonly known as mycobacteria other than tuberculosis (MOTT) may further exacerbate the impact of drug resistance. NTM from various sources have greatest potential for diverse strains among which are resistant strains due to continued evolutional changes.

**Conclusion:**

A collaborative interdisciplinary approach among professionals could help in solving the threats posed by mycobacterial infections to public health, particularly by the spread of drug-resistant strains.

## Background

The One Health (OH) concept is a recent iteration of the One Medicine concept that was first established in the 19th and 20th centuries using the term coined by Calvin W. Schwabe (1927–2006) [1]. The OH concept was introduced as a role model in disease surveillance through an integrated approach adopted by medical, veterinary, and environmental practitioners. OH is a deliberate attempt to deviate from the traditional, narrow-disciplinary approach, to a more holistic, integrated approach that requires multidisciplinary input [2] and serves as a tool in solving complex issues such as food safety, microbial resistance to antibiotics, climate change, and wildlife conservation [3]. This is particularly important in low-income countries, where resources are limited, but there is a vast array of issues to address.

The OH concept gained momentum in 2003 during the avian influenza pandemic threat (HPAI-H5N1) that killed 339 people and caused a global economic loss of an estimated USD 20 billion [4]. The lessons learnt from the HPAI-H5N1 pandemic called for professional collaboration in response to emerging and re-emerging infectious diseases at local and international levels [3]. The Southern African Centre for Infectious Disease Surveillance (SACIDS), a regional consortium of academic and research institutions in Eastern Africa, in partnership with academic institutions in South Africa and the United Kingdom, focuses on the OH concept in response to emerging and re-emerging infectious diseases at local and international levels [3]. The African continent faces a threat from emerging or re-emerging pathogens, infectious diseases of epidemic nature that may occur in endemic form, and the persistence of endemic tropical diseases that are often neglected. Furthermore, considering that the majority of infectious diseases in humans can be traced to an animal origin, Rweyemamu et al. [5, 6] have advocated OH as the most cost-effective approach for the risk management of infectious diseases in Africa. Thus, the concept of OH is clearly applicable to mycobacterial infections that have gravely threatened human and animal health at a global level.

This review seeks to address the mycobacterial infection and challenges associated with its control and diagnosis through One Health approach. Further, it highlights the disease situation, lesson learnt and future perspectives in the control and prevention of mycobacterial infection in Tanzania.

## The OH approach in surveillance of mycobacterial infections in Tanzania

Disease surveillance using the OH approach has recently been a subject of immense interest due to the emergence of infectious diseases that might be caused by close proximity between animals and humans. The genus *Mycobacterium*, consisting of more than 150 well-characterized species, infects both humans and animals [1, 7, 8]. All the members of this genus appear similar on staining for the detection of acid-fast bacilli (AFB) [8], are aerobic, non-spore formers, non-motile, and rod shaped [9, 10]. Mycobacterial infections have received a great attention due to their ubiquitous distribution and ability to infect a wide range of hosts [1]. Their widespread nature and importance in public health emphasize the need for information sharing and active collaboration between experts from a variety of disciplines [1]. The tuberculous mycobacteria (*M. tuberculosis* complex), comprising of *M. tuberculosis*, *M. bovis*, *M. bovis* BCG, *M. canettii*, *M. africanum*, *M. pinnipedii*, *M. microti*, *M. caprae*, (the dassie and the oryx bacillus), and the recently discovered *M. mungi*, are known to be causative agents of tuberculosis in animals and humans. However, non-tuberculous mycobacteria (NTM), also known as environmental mycobacteria, have gained importance in recent years due to the emergence of the Human Immunodeficiency Virus (HIV). Other mycobacterial species, including *M. leprae*, *M. ulcerans*, and *M. paratuberculosis*, also pose a threat to public health.

The public health importance of the genus *Mycobacterium* is based on its role as the causative agent of zoonotic diseases, including tuberculosis in animals (bovine tuberculosis) and humans. Katale et al. [11] tested the hypothesis as to whether the close proximity between humans and animals might have contributed to the cross-species transmission of tuberculosis. This hypothesis was driven by anthropogenic changes, which might have contributed to disease emergence at human-animal interface areas. Changes in the flow of pathogens due to anthropogenic changes have direct and indirect effects on the numbers of susceptible or exposed individuals, or cause increased infectivity [12]. Recently, studies in the human-animal interface areas in Tanzania have reported a relatively low prevalence of bovine tuberculosis (bTB) in indigenous cattle [11, 13]. However, despite the low prevalence of bTB in these interface areas, the authors predicted possibilities for the cross-species transmission of bTB among the interacting hosts, because of poor knowledge among livestock keepers on the transmission dynamics of bTB between wildlife, livestock, and humans [11].

*M. tuberculosis* is primarily a human pathogen with a potential for infecting a wide range of hosts, including wild animals [14, 15] and livestock [16, 17]. In a cross-sectional study of tuberculosis infection that used the OH approach at the human-livestock-wildlife interface of the Serengeti ecosystem, the analysis of the genotype and phylogeographic distribution of *M. tuberculosis* strains isolated from humans revealed a variety of *M. tuberculosis* strains with the predominance of a few successful genotypes, namely, the Central-Asian-strain (CAS), T, Latin-American-Mediterranean (LAM) and East-African-Indian (EAI) families, indicating unlinked transmission chains [18]. These strains were thought to result from a gradually evolving *M. tuberculosis* population, rather than from imported strains [19]. This selective predominance of *M. tuberculosis* strains in Tanzania seems to co-exist with variations depending on the location of the samples within Tanzania. In the Tanga region of northern Tanzania, the EAI and CAS family genotypes appear to be predominant [20]. Furthermore, Eldholm et al. [19] reported that the human TB epidemic, that which is caused by a few successful *M. tuberculosis* families, is dominated by the CAS family in Dar es Salaam. Other TB strains recorded were LAM (22%) and EAI (17%). Beijing and T-family genotypes, as well as importation of strains, were suggested to be a minor problem. Nevertheless, despite the dominance of the CAS strain in Dar es Salaam and Tanzania in general, there were variations in the TB strains within *M. tuberculosis* families [19]. Although the Beijing lineage of *M. tuberculosis* has been reported to be found in low proportion [18, 19], its presence is of major concern as it has been said to be evolutionarily more associated with drug resistance [21, 22] and presents severe disease symptoms compared to the other lineages [23]. In addition, the LAM-TB family genotype, which has also been found in Tanzania, has been associated with drug resistance that can be attributed to the genetic background of particular strains favoring drug resistance or pre-existing disproportionate exposure to TB drugs of a particular TB family genotype [24]. The challenge with drug resistance is the fact that the genetics of observed drug resistance is more complex than previously expected [25].

Nearly all the TB strains identified all over the world have been isolated in East Africa [18], an indication of historical migrations that have occurred during the pre- and post-colonial rule. Historical movements due to international trade, increased movement of wild and domestic animals, and their interaction has contributed to the global spread of pathogenic organisms such as *M. tuberculosis* at an accelerated rate [1, 26, 27]. Likewise, the diversity of *M. tuberculosis* strains in different regions within Tanzania might be attributed to a cosmopolitan population with frequent migration and travel [18, 20]. This spread could also be explained by the ancestral Afro-Asian trade networks existing from a long time [28].

*M. bovis* is a multi-host pathogen capable of infecting a wide range of hosts including humans [29]. Such broad-spectrum pathogens tend to pose a greater epidemiological threat than the more specialized ones [30]. In Tanzania, studies on *M. bovis* infections in animals have been conducted using the single comparative intradermal tuberculin test (SCITT) [11, 13, 31–35], gamma interferon assay [36], and molecular diagnostic techniques such as spacer oligotyping, mycobacterium interspersed repetitive units-variable number of tandem repeats (MIRU-VNTR) [13, 37, 38], and competitive enzyme-linked immunoassay (ELISA) [39]. The molecular characterization of *M. bovis* isolated from the human-animal interface areas indicated no clear evidence for recent cross-species transmission of *M. bovis* between humans, livestock, and wild animals in Tanzania [37]. However, it was observed that bTB infections in wild animals and cattle were epidemiologically related [37]. In a recent study, Katale et al. [37] reported that *M. bovis* isolates belonging to the SB0133 spoligotype isolated from wildlife had 45.2 and 96.8% spoligotype pattern agreement with novel SB2290 and SB2289 strains from indigenous cattle, respectively [37]. The SB2290 novel spoligotype isolated from indigenous cattle differed from spoligotypes found in buffalo and African civet by the loss of a single spacer, indicating the epidemiological relationship of *M. bovis* at the livestock-wildlife interface. This association could mean that wild animals acquire *M. bovis* infection from domestic animals rather than vice versa, as strains with more spacers are evolutionarily older than those with few spacers. Moreover, it was suggested that there was a spillback of *M. bovis* infection from wild animal reservoirs to livestock, or micro-evolutionary events of *M. bovis* in cattle populations in the ecosystem. It could also be argued that the observed genetic structure of *M. bovis* resulted from evolutionary events taking place in cattle populations outside the study area, possibly where the prevalence of the disease was higher and that the *M. bovis* strains were merely imported into cattle and wild animal populations in the Serengeti ecosystem [37]. Therefore, wild animals in their ecosystems were at risk of acquiring *M. bovis* infection due to occasional interactions such as sharing of pasture and water sources with livestock. Similarly, livestock could acquire infections from wild animals that may be reservoirs of certain transmissible diseases [13, 40]. In 2013, Mwakapuja and colleagues found SB1467 to be the dominant spoligotype isolated from indigenous cattle at the livestock/wildlife interface areas in the Morogoro region of eastern Tanzania [13]. The authors also found that the SB2190 novel spoligotype isolated from indigenous cattle was 59.4% related to SB0133, which was the predominant spoligotype in the East African countries.

Previous studies conducted in Tanzania had have reported a variety of atypical mycobacteria in culture isolates from humans [41, 42] and animals [43, 44], indicating the diversity of NTM species, some of which were capable of causing diseases in animals and humans. The differences in species distribution may partly determine the frequency and manifestation of pulmonary NTM disease at each geographical location [45]. In Tanzania, several NTM species, including *M. gordonae, M. interjectum, M. intracelullare, M. sherrisii, M. avium*. spp.*,* and *M. fortuitum* have been found in humans [20, 41, 46], while *M. simiae, M. confluentis, M. neoaurum, M. nonchromogenicum, M. terrae, M. thermoresistibile, M. genavense, M. gilvum, M. intermedium, M. poriferae, M. spaghni, M. kansasii, M. gastri, M. indicus pranii, M. hibernae, M. engbaekii, M. septicum, M. arupense, M. peregrinum, M. moriokaense, M. palustre, M. goodie, M. gordonae, M. smegmatis, M. fortuitum, M. phlei, M. flavescens*, and *M. avium intracellulare* have been isolated from indigenous cattle [34, 41, 44] and *M. lentiflavum* and *M. intracellulare* have been reported in wildlife species [41]. However, Hoefsloot et al. [45] reported that mycobacteria of the *M. avium* complex (MAC) were predominant in most of the countries, followed by *M. gordonae* and *M. xenopi*. Mwikuma et al. [47] also found a diverse range of NTM species with a predominance of *M. intracellulare,* which causes disease in immunocompromised as well as immunocompetent subjects [48]. The members of NTM induce progressive pulmonary diseases in older persons, superficial lymphadenitis, disseminated disease in severely immunocompromised patients, and skin and soft tissue infections [49]. In northern Tanzania, lymphadenitis in TB infections due to NTM was reported in 31 (47.7%) patients, compared to 7 (10.8%) *M. bovis* patients and 27 (41.5%) *M. tuberculosis* patients [50]. It is worth mentioning that some of the NTM have potential health devastating effects [46], both in healthy and immunocompromised individuals. For example, invasive NTM infections due to *M. sherrisii* and *M. avium* complex sequevar *M. avium* complex-D have been diagnosed in HIV-infected patients in northern Tanzania [46].

In Tanzania, *M. bovis* has been isolated from human in cases of extrapulmonary tuberculosis [38, 50], livestock [13, 37, 51] and wildlife [37, 39], signifying threat of transmission of mycobacteria between livestock, wildlife and humans in the country. For instance, Mfinanga and colleagues found high proportion of atypical mycobacteria (31 (47.7%) in human as compared to 7 (10.8%) *M. bovis*, and 27 (41.5%) *M. tuberculosis* [50]. Further, Kazwala et al. [51], in their study in Mbeya region, Southern Highland Tanzania, investigated a total of 31 *M. bovis* isolate from cattle and five isolates from human, of which there was evidence of overlap between DNA fingerprints of *M. bovis* between cattle and human. However, the control and prevention of zoonotic infections including mycobacteria possess a challenge due to weak surveillance systems in our local settings attributed by lack of policies harmonization and limited resources for diseases control. Therefore, there is need for synergy of veterinary and medical policies in the control of tuberculosis in our local settings [51] to optimize the efforts to ensure there is a better response to disease threats. Further, governments should increase allocation of funds for surveillance, control and prevention of infectious zoonotic diseases to safeguard health for animals and humans.

## Diagnosis and challenges associated with mycobacterial infections

The accurate diagnosis of mycobacterial species is complicated and an ongoing problem that has passed through a number of stages, from the testing of drug susceptibility in the mid–1980s, to use of nucleic-acid probes in the late 1980s, nucleic acid amplification (NAA) in the mid–1990s, and DNA sequencing at present [8]. The recently introduced molecular techniques are based on NAA tests that are used directly on clinical specimens and complemented by blood tests (QuantiFERON-TB, T-SPOT.TB test) that measure the IFN-γ released by stimulated T cells. These techniques reduce the time frame for TB diagnosis from weeks to days [52]. These newer molecular methods provide complementary information to conventional diagnostic techniques based on culture and microscopy, thus improving patient management [52].

The conventional microscopic examination, culture, and drug susceptibility testing (DST) of sputum samples are the most common diagnostic techniques for the detection of the genus *Mycobacterium*. In many countries, microscopic examination techniques help in the detection of *M. tuberculosis*, culture-based methods are the cornerstone for diagnosis of TB, and detection of drug resistant strains is the simplest method for the presumptive diagnosis of TB [53]. However, the conventional microscopic test, which detects the presence of acid-fast bacilli (AFB), is not useful for the identification of species of the genus *Mycobacterium*. Therefore, there is an urgent need for highly sensitive, specific, and rapid diagnostic techniques that can be performed at the point of care for the identification of *M. tuberculosis* and NTM disease. The WHO emphasizes TB disease to be resistant to pyrazinamide - one of the standard first-line medications used to treat TB with risk for patients being often misdiagnosed and receiving ineffective treatment being not uncommon. As such WHO proposes advocacy on developing strategies to improve food safety, developing capacity of the animal health sector to reduce the prevalence of TB in livestock and identification of key populations and risks pathways for transmission of zoonotic TB to break the chain of transmission [54]. Some NTM are relatively resistant to several of the first- and second-line TB drugs, thus making the accurate diagnosis and drug-susceptibility testing critical for the clinical management of NTM infection [55]. Timely and accurate identification of TB and NTM diseases could influence both therapy and epidemiology of TB and TB-like diseases [20]. The recent advent of whole genome sequencing (WGS) has improved our understanding of the transmission dynamics and identification of mycobacterial infections as well as their drug resistance mutations, which helps in early identification of the resistance profile of the infecting strain [53]. However, the use of WGS in developing countries is limited by factors such as high running costs and the possibility of its accommodation into pre-existing diagnostic frameworks [56]. Thus, several barriers such as high diagnostic costs and the absence of automated sequence analysis pipelines and supporting IT infrastructure limit the widespread adoption of WGS in developing countries [57].

Infections with mycobacteria are subjected to other challenges, including co-infection with other pathogens, development of drug resistance, and increased incidences of NTM infections both in immunocompetent and immunocompromised individuals. Global incidences of mycobacterial infections have increased in recent decades due to the Human Immunodeficiency Virus (HIV) pandemic. Co-infection by HIV and TB accelerates the decline of immunological functions, leading to subsequent death if left untreated [58]. HIV infection does not only increase susceptibility to TB but also predispose infection to NTM which further complicate TB diagnosis. Infections with NTM in AIDS patients are associated with increased morbidity and high rates of mortality [59]. In spite of access to active antiretroviral therapy (ART) for AIDS patients, the concurrent management of HIV/TB co-infection remains a challenge, due to adverse effects, drug interactions, and toxicities [60]. Conversely, the mechanisms leading to the breakdown of the immune defense in HIV-TB co-infected individuals are not well described [58, 59].

## Lessons learnt and future perspective of OH initiatives in Tanzania

Disease epidemics of zoonotic nature including other agents than mycobacteria, can better be controlled and managed through joint OH multidisplinary approach. This has been learnt in epidemics such as Anthrax, Ebola, rabies and Rift Valley fever outbreaks where various key player involvement is necessary to put the situation under custody. The prevention and control of infectious diseases depend on the early recognition of the causative agents and a prompt response. Recently, a number of initiatives to address the various aspects of infectious disease in humans and animals have been established in Tanzania. For example, the SACIDS has been a role model for the surveillance of a variety of infectious diseases, including mycobacterial infections. It is worth noting that other OH initiatives such as One Health Central and Eastern Africa (OHCEA) and AfriqueOne have supported research activities that also focus on infectious diseases of humans and animals in Tanzania. Therefore, to advocate for the OH approach in disease surveillance, intersectoral collaboration among stakeholders, government sectors, and society are necessary to address the factors of health and well-being of humans and animals [61]. In East Africa, strengthening of the OH networks has been marked by the establishment of OH coordination units in the respective countries. This move has gained momentum due to the status of the East African countries as risk areas for infectious diseases such as Rift Valley Fever and avian influenza, as well as the detection of Ebola virus in the neighboring Democratic Republic of Congo (DRC). These OH coordination units will act as a bridge between the OH networks and their respective government ministries. For example, the OH unit established under ‘Disaster Management’ in the Prime Minister’s office in Tanzania will serve as a platform wherein all matters related to OH activities will be coordinated at all levels to ensure smooth communication and a rapid response by professionals during periods of epidemic and inter-epidemic. This is similar to Kenya, where the Zoonotic Unit linking the human and animal health departments is in place under the Ministries of Health and Livestock/Agriculture. Similarly, other OH networks have been established in countries such as India and South Africa, indicating acceptance of the OH concept in low and middle income countries [1]. Various regional disease surveillance programmes have been established for the joint control of shared disease calamities. For example, regional infectious disease surveillance bodies, such as the Connecting Organizations for Regional Disease Surveillance (CORDS), were established in 2010 to improve the interactions between members and other global partners in order to strengthen international health security.

Our experience shows that the OH approach brings healthcare professionals to a common platform that could improve cooperation during the surveillance of diseases. This approach could facilitate the implementation of objectives of OH, through improvement of the status of the education system, administrative structures, and legislation [62]. Thus, the infrastructure, as well as capacity of veterinary and human health facilities, as well as capacity of facilities should be strengthened to facilitate the exchange of information between the two sectors. In addition, the private sector should not be side-lined, but rather should be considered a partner in sharing the costs in proportion to the benefits, when distributing responsibility for emerging pathogens. A smart partnership of SACIDS and local institutions in Tanzania with external institutions such as the London School of Hygiene and Tropical Medicine (LSHTM) and the Royal Veterinary College (RVC) in the United Kingdom, and with the University of Pretoria and Stellenbosch University in South Africa, has brought together academicians and researchers to collaborate on supervision and research activities. This has enabled young researchers to utilize both local and global facilities for research. The SACIDS, in collaboration with the Sokoine University of Agriculture (SUA), Muhimbili University of Health and Allied Sciences (MUHAS), the National Institute for Medical Research (NIMR), and the Tanzania Wildlife Research Institute (TAWIRI), has supported the OH approach and encouraged the shared use of both veterinary and human health facilities including laboratories. Training modules on OH concepts have been established in local universities to impart inter-disciplinary training on OH to young scientists. The complex nature of zoonotic diseases and limited resources in developing countries are reminders of the need for the implementation of OH in disease surveillance in low-resource settings [63]. Cost-effective disease surveillance should be implemented to ensure that the few resources that are available are streamlined and synergistically developed between human, animal, and environmental health in order to prioritize disease control programs.

## Conclusions

In conclusion, the zoonotic importance of mycobacterial infection and the possibility of co-infections with other pathogens highlights the need for collaborative efforts among professionals in terms of sharing of research and resources, to ensure cost-effective control of diseases. Therefore, it is important to establish consistent communication among professionals in order to undertake joint actions toward the prevention and control of emerging zoonotic diseases including mycobacterial infections. Moreover, universities and research institutions should take a lead to change the mindset of young scientists to reduce insularity when it comes to controlling of infectious diseases of public health importance. It is worth noting that synergy among medical, veterinary, and environmental professionals in the surveillance of diseases could reduce cost and improve information sharing. However, the main concern is whether these professionals are willing to work on a common platform, something that might necessitate a change of attitude in order to achieve the best outcome.

## Data Availability

The whole information provided in this paper was generated from peer reviewed published articles.

## References

[1] Thirunavukkarasu S, Plain KM, de Silva K, Marais BJ, Whittington RJ (2017). Applying the one health concept to mycobacterial research–overcoming parochialism. Zoonoses Public Health.

[2] Travis DA, Chapman DW, Craft ME, Deen J, Farnham MW, Garcia C, Hueston WD, Kock R, Mahero M, Mugisha L et al.: One Health: lessons learned from East Africa. Microbiol Spectrum 2014, 2 (1):OH–0017–2012. 10.1128/microbiolspec.OH-0017-2012 26082115

[3] Gibbs EPJ (2014). The evolution of one health: a decade of progress and challenges for the future. Vet Rec.

[4] FAO: H1N5, Global Overview. file:///C:/Users/Lenovo/Desktop/Folders/Review/OH-References/FAO–2012.pdf. 2014.

[5] Rweyemamu MM, Paweska J, Kambarage D, Namuba F: Toward One Africa, One Health: the SACIDS One Health focus on infectious diseases. . Onderstepoort Vet J 2012, 79 (2):Art. #449, 442 pages. 10.4102/ojvr.v79i2.449 23327368

[6] Rweyemamu M, Kamabarage D, Karimuribo E, Wambura P, Matee M, Kayembe JM, Mweene A, Neves L, Masumu J, Kasanga C et al.: Development of a One Health National Capacity in Africa. In: Mackenzie J., Jeggo M., Daszak P., Richt J. (eds) One Health: The Human-Animal-Environment Interfaces in Emerging Infectious Diseases. . Current Topics in Microbiology and Immunology 2013, 366. Springer, Berlin, Heidelberg. 10.1007/82_2012_244 22820706

[7] Teran R, de Waard JH: Recent advances in the laboratory diagnosis of tuberculosis. Journal of International Federation of Clinical Chemistry and Laboratory Medicine. 26 2015, 4:295–309.

[8] Hale YM, Pfyffer GE, Salfinger M (2001). Laboratory diagnosis of mycobacterial infections: new tools and lessons learned. Clin Infect Dis.

[9] Quinn PJ, Markey BK, Leonard FC, Hartigan P, Fanning S, Fitzpatrick ES: Veterinary Microbiology and Microbial Diseases. John Wiley & Sons, ISBN 1118251164, 9781118251164, 400 pgs, . 2011.

[10] Johnson MM, Odell JA (2014). Nontuberculous mycobacterial pulmonary infections. J Thorac Dis.

[11] Katale BZ, Mbugi EV, van Helden P, Keyyu JD, Kendall S, Kibiki GS, Godfrey-Faussett P, Kazwala RR, Karimuribo ED, Michel A (2013). Prevalence and risk factors for bTB infection in indigenous cattle at human-livestock-wildlife interface of the Serengeti ecosystem, Tanzania. BMC Vet Res.

[12] Lindahl Johanna F., Grace Delia (2015). The consequences of human actions on risks for infectious diseases: a review. Infection Ecology & Epidemiology.

[13] Mwakapuja RS, Makondo ZE, Malakalinga J, Moser I, Kazwala RR, Tanner M (2013). Molecular characterization of Mycobacterium bovis isolates from pastoral livestock at Mikumi-Selous ecosystem in the eastern Tanzania. Tuberculosis (Edinb).

[14] Mikota S, Sargent EL, Ranglack GS. Medical management of the elephant. Tuberc Tuberculin Test. 1994:33–9.

[15] Michalak Kathleen (1998). Mycobacterium tuberculosis infection as a Zoonotic Disease: Transmission between Humans and Elephants. Emerging Infectious Diseases.

[16] Ocepek M, Pate M, Žolnir-Dovč M, Poljak M (2005). Transmission of Mycobacterium tuberculosis from human to cattle. J Clin Microbiol.

[17] Srivastava K, Chauhan DS, Gupta P, Singh HB, Sharma VD, Yadav VS, Sreekumaran, Thakral SS, Dharamdheeran JS, Nigam P et al.: solation of Mycobacterium bovis & M. tuberculosis from cattle of some farms in North India–possible relevance in human health. Indian J Med Res 2008, 128:26–31. 18820355

[18] Mbugi EV, Katale BZ, Siame KK, Keyyu JD, Kendall SL, Dockrell HM, Streicher EM, Michel AL, Rweyemamu MM, Warren RM (2015). Genetic diversity of Mycobacterium tuberculosis isolated from tuberculosis patients in the Serengeti ecosystem in Tanzania. Tuberculosis (Edinb).

[19] Eldholm V, Matee M, Mfinanga S, Heun M, Dahle U (2006). A first insight into the genetic diversity of Mycobacterium tuberculosis in Dar Es Salaam, Tanzania, assessed by spoligotyping. BMC Microbiol.

[20] Hoza AS, Mfinanga SG, Moser I, König B (2016). Molecular characterization of Mycobacterium tuberculosis isolates from Tanga, Tanzania: first insight of MIRU-VNTR and microarray-based spoligotyping in a high burden country. Tuberculosis (Edinb).

[21] Ioerger TR, Feng Y, Chen X, Dobos KM, Victor TC, Streicher EM, Warren RM, van Pittius NCG, Van Helden PD, Sacchettini JC (2010). The non-clonality of drug resistance in Beijing-genotype isolates of Mycobacterium tuberculosis from the Western cape of South Africa. BMC Genomics.

[22] Haeili M, Darban-Sarokhalil D, Fooladi AAI, Javadpour S, Hashemi A, Siavoshi F, Feizabadi MM (2013). Spoligotyping and drug resistance patterns of Mycobacterium tuberculosis isolates from five provinces of Iran. Microbiology Open.

[23] Gygli SM, Borrel S, Trauner A, Gagneux S (2017). Antimicrobial resistance in Mycobacterium tuberculosis: mechanistic and evolutionary perspectives. FEMS Microbiol Rev.

[24] Grandjean L, Iwamoto T, Lithgow A, Gilman RH, Arikawa K, Nakanishi N, Martin L, Castillo E, Alarcon V, Coronel J (2015). Association between Mycobacterium tuberculosis genotype and drug resistance in Peru. PLoS One.

[25] Zhang H, Li D, Zhao L, Fleming J, Lin N, Wang T, Liu Z, Li C, Galwey N, Deng J (2013). Genome sequencing of 161 Mycobacterium tuberculosis isolates from China identifies genes and intergenic regions associated with drug resistance. Nat Genet.

[26] Cohen M (2000). Changing patterns of infectious disease. Nature.

[27] Rabinowitz P, Scotch M, Conti L (2009). Human and animal sentinels for shared health risks. Vet Ital.

[28] Mbugi EV, Katale BZ, Streicher EM, Keyyu JD, Kendall SL, Dockrell HM, Michel ALM, Rweyemamu MM, Warren RM, Matee MI (2016). Mapping of Mycobacterium tuberculosis complex genetic diversity profiles in Tanzania and other African countries. PLoS One.

[29] Renwick A, White P, Bengis R (2007). Bovine tuberculosis in southern African wildlife: a multi-species host-pathogen system. A review. Epidemiol Infect.

[30] McCallum H, Dobson A (1995). Detecting disease and parasite threats to endangered species and ecosystems. Trends Ecol Evol.

[31] Kazwala RR, Kambarage DM, Daborn CJ, Nyange J, Jiwa SFH, Sharp JM (2001). Risk factors associated with the occurrence of bovine tuberculosis in cattle in the southern highlands of Tanzania. Vet Res Commun.

[32] Jiwa SFH, Kazwala RR, Aboud AAO, Kalaye WJ (1997). Bovine tuberculosis in the Lake Victoria zone of Tanzania and its possible consequences for human health in the HIV/AIDS era. Vet Res Commun.

[33] Shirima GM, Kazwala RR, Kambarage DM (2003). Prevalence of bovine tuberculosis in cattle in different farming system in Tanzania. Prev Vet Med.

[34] Mdegela RH, Kusiluka LJM, Kapaga AM, Karimuribo ED, Turuka FM, Bundala A, Kivaria F, Kabula B, Manjurano A, Loken T (2004). Prevalence and determinants of mastitis and Milk-borne Zoonoses in smallholder dairy farming sector in Kibaha and Morogoro districts in eastern Tanzania. JVet Med B.

[35] Durnez L, Sadiki H, Katakweba A, Machang’u RR, Kazwala RR, Leirs H, Portaels F (2009). The prevalence of Mycobacterium tuberculosis and atypical mycobacterioses in cattle in and around Morogoro, Tanzania. Trop Anim Health Prod.

[36] Katale BZ, Fyumagwa RD, Eblate EM, Kuya S, Batamuzi EK, Matee MI, Keyyu JD, Muumba J, Mdaki M, Mbugi EV, et al. Screening of bovine tuberculosis in African buffaloes in Ngorongoro Conservation Area, Northern Tanzania: Implication for public health. J Wildl Dis. 2017. 10.7589/2016-7510-7223. 10.7589/2016-10-223 28657858

[37] Katale BZ, Mbugi EV, Siame KK, Keyyu JD, Kendall S, Kazwala RR, Docrell HM, Fyumagwa RD, Michel AL, Rweyemamu M (2015). Isolation and potential for transmission of Mycobacterium bovis at human-livestock-wildlife interface of the Serengeti ecosystem, Northern Tanzania. Transbound Emerg Dis.

[38] Kazwala RR, Kusiluka LJM, Sinclair K, Sharp JM, Daborn CJ (2006). The molecular epidemiology of Mycobacterium bovis infections in Tanzania. Vet Microbiol.

[39] Cleaveland S, Mlengeya T, Kazwala RR, Michel A, Kaare MT, Jones SL, Eblate E, Shirima GM, Packer C (2005). Tuberculosis in Tanzanian wildlife. J Wildl Dis.

[40] Musoke J, Hlokwe T, Marcotty T, du Plessis BJA, Michel AL (2015). Spillover of Mycobacterium bovis from wildlife to livestock, South Africa. Emerg Infect Dis.

[41] Katale BZ, Mbugi EV, Botha L, Keyyu JD, Kendall S, Dockrell HM, Michel AL, Kazwala RR, Rweyemamu MM, van Helden P (2014). Species diversity of non-tuberculous mycobacteria isolated from humans, livestock and wildlife in the Serengeti ecosystem, Tanzania. BMC Infect Dis.

[42] Hoza AS, Mfinanga SGM, Rodloff AC, Moser I, Konig B (2016). Increased isolation of nontuberculous mycobacteria among TB suspects in northern Tanzania: public health and diagnostic implications for control programmes. BMC Res Notes.

[43] Kaneene JB, Miller R, Kaplan B, Steele JH, Thoen CO (2014). Preventing and controlling zoonotic tuberculosis: a one health approach. Vet Ital.

[44] Makondo ZE, Kazwala RR, Mwakapuja RS, Malakalinga J, Moser I, Tanner M (2014). Nontuberculous mycobacteria infections in Katavi Rukwa ecosystems. J Agric Sci Technol B.

[45] Hoefsloot W, van Ingen J, Andrejak C, Angeby K, Bauriaud R, Bemer P, Beylis N, Boeree MJ, Cacho J, Chihota V (2013). Nontuberculous mycobacteria network European trials group. The geographic diversity of nontuberculous mycobacteria isolated from pulmonary samples: an NTM-NET collaborative study. Eur Respir J.

[46] Crump JA, van Ingen J, Morrissey AB, Boeree MJ, Mavura DR, Swai B, Thielman NM, John AB, Grossman H, Maro VP (2009). Invasive disease caused by Nontuberculous mycobacteria, Tanzania. Emerg Infect Dis.

[47] Mwikuma G, Kwenda G, Hang’ombe BM, Simulundu E, Kaile T, Nzala S, Seter Siziya S, Suzuki Y (2015). Molecular identification of non-tuberculous mycobacteria isolated from clinical specimens in Zambia. Ann Clin Microbiol Antimicrob.

[48] Han XY, Tarrand JJ, Infante R, Jabson KL, Truong M (2005). Clinical significance and epidemiologic analyses of Mycobacterium avium and Mycobacterium intracellulare among patients without AIDS. J Clin Microbiol.

[49] Griffith DE, Aksamit T, Brown-Elliott BA, Catanzaro A, Daley C, Gordin F, Holland SM, Horsburgh R, Huitt G, Iademarco MF (2007). ATS mycobacterial diseases subcommittee; American Thoracic Society; infectious disease Society of America. An official ATS/IDSA statement: diagnosis, treatment, and prevention of non-tuberculous mycobacterial diseases. Am J Respir Crit Care Med.

[50] Mfinanga SGM, Morkve O, Kazwala RR, Cleaveland S, Sharp MJ, Kunda J, Nilse R (2004). Mycobacterial adenitis, role of Mycobacterium bovis, non tuberculous mycobacteria, HIV infection, and risk factors in Arusha, Tanzania. East Afr Med J.

[51] Kazwala RR, Daborn CJ, Kusiluka LJM, Jiwa SFH, Sharp JM, Kambarage DM (1998). Isolation of Mycobacterium species from raw milk of pastoral cattle of the southern highlands of Tanzania. Trop Anim Health Prod.

[52] Teran R, de Waard JH (2015). Recent advances in the laboratory diagnosis of tuberculosis. J Int Fed Clin Chem Lab Med.

[53] Palomino Juan Carlos, Martin Anandi (2015). Challenges Associated with Diagnostics, Drug Resistance, and Pathogenesis of Mycobacterium Tuberculosis. Human Emerging and Re-emerging Infections.

[54] WHO, 2017. WHO Zoonotic TB Roadmap, 2017. “https://www.who.int/tb/features_archive/zoonotic_TB_roadmap/en/. Accessed 15 July 2018.

[55] Wang X, Li H, Jiang G, Zhao L, Ma Y, Javid B, Huang B (2014). Prevalence and drug resistance of Nontuberculous mycobacteria, northern China, 2008–2011. Emerg Infect Dis.

[56] Mohamed S, Naidoo K, Dookie N, Padayatchi N (2017). Whole genome sequencing for the management of drug-resistance TB in low income high TB burden settings: challenges and implications. Tuberculosis (Edinb).

[57] Pankhurst LJ, del Ojo EC, Votintseva AA, Walker TM, Cole K, Davies J, Fermont JM, Gascoyne-Binzi DM, Kohl TA, Kong C (2016). Rapid, comprehensive, and affordable mycobacterial diagnosis with whole-genome sequencing: a prospective study. Lancet Respir Med.

[58] Pawlowski A, Jansson M, Sköld M, Rottenberg ME, Källeniu G (2012). Tuberculosis and HIV co-infection. PLoS Pathog.

[59] Thomsen VØ, Dragsted UB, Bauer J, Fuursted K, Lundgren J (1999). Disseminated infection with Mycobacterium genavense: a challenge to physicians and Mycobacteriologists. J Clin Microbiol.

[60] Shankar EM, Vignesh R, Ellegård R, Barathan M, Chong YK, Bador MK, Rukumani DV, Sabet NS, Kamarulzaman A, Velu V (2014). HIV-Mycobacterium tuberculosis co-infection: a ‘danger-couple model’ of disease pathogenesis. Pathog Dis.

[61] WHO: Health Equity Through Intersectoral Action: An Analysis of 18CountryCaseStudies .http://www.who.int/social_determinants/resources/health_equity_isa_2008_en.pdf?ua=1. Accessed 15 July 2018.

[62] Aliyi S, Birhanu T, Gizachew A, Kebeta T (2015). One health program: its future implications, Challenges and Opportunities: Review. Nat Sci.

[63] Gebreyes WA, Dupouy-Camet J, Newport MJ, Oliveira CJB, Schlesinger LS, Saif YM, Kariuki S, Saif LJ, Saville W, Wittum T (2014). The global one health paradigm: challenges andOpportunities for tackling infectious diseases at the human, animal, and environment Interface in low-resource settings. PLoS Negl Trop Dis.

